# Microstructure Study of Pulsed Laser Beam Welded Oxide Dispersion-Strengthened (ODS) Eurofer Steel

**DOI:** 10.3390/mi12060629

**Published:** 2021-05-28

**Authors:** Jia Fu, Ian Richardson, Marcel Hermans

**Affiliations:** 1 Department of Materials Science and Engineering, Delft University of Technology, 2628 CD Delft, The Netherlands; I.M.Richardson@tudelft.nl (I.R.); M.J.M.Hermans@tudelft.nl (M.H.); 2 Dutch Institute for Fundamental Energy Research (DIFFER), 5600 HH Eindhoven, The Netherlands

**Keywords:** oxide dispersion strengthened steel, ODS Eurofer, laser welding, microstructure, EBSD

## Abstract

Oxide dispersion-strengthened (ODS) Eurofer steel was laser welded using a short pulse duration and a designed pattern to minimise local heat accumulation. With a laser power of 2500 W and a duration of more than 3 ms, a full penetration can be obtained in a 1 mm thick plate. Material loss was observed in the fusion zone due to metal vaporisation, which can be fully compensated by the use of filler material. The solidified fusion zone consists of an elongated dual phase microstructure with a bimodal grain size distribution. Nano-oxide particles were found to be dispersed in the steel. Electron backscattered diffraction (EBSD) analysis shows that the microstructure of the heat-treated joint is recovered with substantially unaltered grain size and lower misorientations in different regions. The experimental results indicate that joints with fine grains and dispersed nano-oxide particles can be achieved via pulsed laser beam welding using filler material and post heat treatment.

## 1. Introduction

Due to their good high-temperature strength, corrosion resistance and radiation resistance, oxide dispersion-strengthened (ODS) steels are promising candidates for structural materials employed in elevated-temperature and nuclear applications [1]. The favourable properties of ODS steels are mainly attributed to the fine grains and homogenously dispersed nanosized oxide particles in the steel matrix [2]. These fine and thermally stable dispersoids hinder the motion of dislocations and grain boundaries, acting as trapping sites for both point defects and helium atoms generated during irradiation, resulting in an increased resistance to irradiation damage.

Despite the promising behaviour of ODS steels for use in advanced nuclear systems, joining these materials remains one of the major technological challenges limiting their deployment [3]. Joining ODS steels by solid-state methods such as spark plasma sintering (SPS), hot isostatic pressing (HIP) and friction stir welding (FSW) has been proven to be feasible by several authors [4,5,6]. The degradation of featured microstructures and mechanical properties can be minimised since these techniques do not create a molten zone in the joint area [5]. However, the costs of SPS and HIP are relatively high due to long processing times (1–5 h) [7] and the application of FSW is limited due to geometrical restrictions and tool wear [8]. The welding of ODS steels by traditional, fusion-based welding techniques such as gas metal arc welding and tungsten inert gas welding is problematic. As soon as a molten zone is produced, the oxide particles rapidly agglomerate and float to the top of the molten weld pool, resulting in a significant loss of strength [9]. Laser beam welding [10,11,12] can potentially be employed for joining ODS steels due to its highly concentrated energy input, leading to the melting of a small amount of base material, and consequently, the formation of a small heat-affected zone (HAZ). The study of Lemmen et al. [12] showed that PM1000 had a good laser weldability with a wide range of welding parameters. However, yttrium oxide clustering was found in all conditions, causing a reduction in strength in the weld. Similar results were obtained by Liang et al. [13] who indicated that the nanoprecipitates were larger in the weld metal than in the base material. In summary, a new laser welding method needs to be developed to address the issue of microstructure and mechanical behaviour degradation.

In this study, pulsed laser beam welding was successfully employed to join ODS Eurofer steel with only minor deterioration of microstructure when compared to the parent material. The welding parameters were investigated and optimised to improve the microstructure of the joint. The microstructural features were characterised by means of optical microscopy (OM), scanning electron microscopy (SEM), transmission electron microscopy (TEM) and electron backscattered diffraction (EBSD).

## 2. Experimental Details

### 2.1. Materials

**Figure 1:**
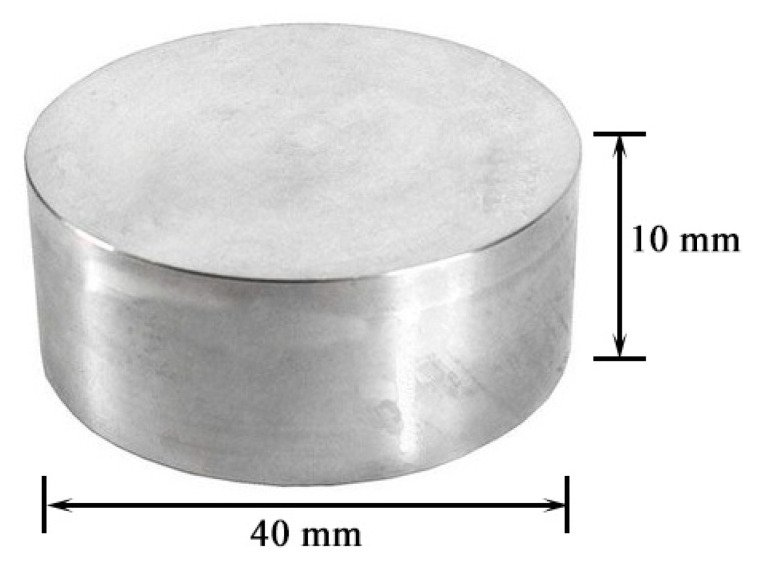
Powder metallurgy-produced ODS Eurofer.

The alloy studied was ODS Eurofer steel with a nominal composition of Fe–9Cr–1.1W–0.4Mn–0.2V–0.12Ta–0.1C–0.3Y_2_O_3_ (wt%), produced via powder metallurgy. The production process started with mechanical alloying, where the precursor powders were mixed in a Retsch planetary ball milling machine under an argon atmosphere for 30 h at 300 rpm. The resultant powders were subsequently consolidated by spark plasma sintering (SPS, FCT group, Frankenblick, Germany) at a pressure of 60 MPa and a sintering temperature of 1373 K with a heating rate of 100 K/min. After a holding time of 30 min, disks of 40 mm diameter and around 10 mm thickness were produced (Figure 1). These parameters were selected based on our previous study [14].

### 2.2. Methods

**Figure 2:**
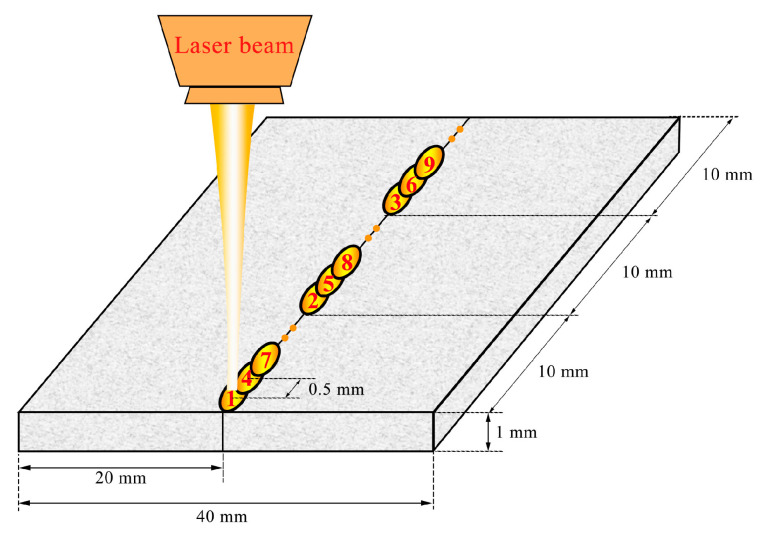
Schematic illustration of welding strategies.

A 6 kW Yb:YAG laser was used for the welding experiments. The focusing optic has a focal length of 223 mm and projects a laser spot with a diameter of 0.6 mm. Two work pieces (30 × 20 × 1 mm^3^) machined from the SPS-prepared disk were welded by pulsed laser beam welding with a peak power of 2500 W and a pulse duration ranging from 2 ms to 5 ms. A shielding gas of argon was delivered to the work piece at a flow rate of 8 L/min. Instead of moving straight in one direction, the laser beam was moved following the sequence indicated in Figure 2 in order to minimise the heat accumulation in the material and, therefore, shorten the melt pool lifetime. The time interval between each point is around 30 s. The distance between the centres of adjacent spots was 0.5 mm to ensure a continuous weld. A post heat treatment was conducted to recover the microstructure and release the residual stress generated during welding by normalising at 1423 K for 1 h, air cooling to room temperature, and then, tempering at 973 K for 1 h, followed by air cooling to room temperature.

The microstructure of the joint was characterised using a Keyence Digital Microscope VHX-5000 and a JEOL 6500F SEM equipped with an energy dispersive spectrometer (EDS) and EBSD. The nano-oxide particles in the material were investigated using a JEM-2200FS TEM. To reveal the microstructure by OM and SEM, the samples were etched in a solution of 5 g ferric chloride, 50 mL HCl and 100 mL distilled water for 20 s. Specimens for EBSD were mirror polished followed by a colloidal silica polishing step. TSL orientation imaging microscopy (OIM) software was used for data processing and analysis. EBSD maps of inverse pole figure (IPF), grain average image quality (GAIQ) and kernel average misorientation (KAM) were implemented and analysed in the study. The TEM specimens were prepared by electropolishing disks with a diameter of 3 mm in a twin-jet electropolisher using 4% perchloric acid and 96% ethanol as electrolyte.

## 3. Results

### 3.1. Parameter Optimisation

**Figure 3:**
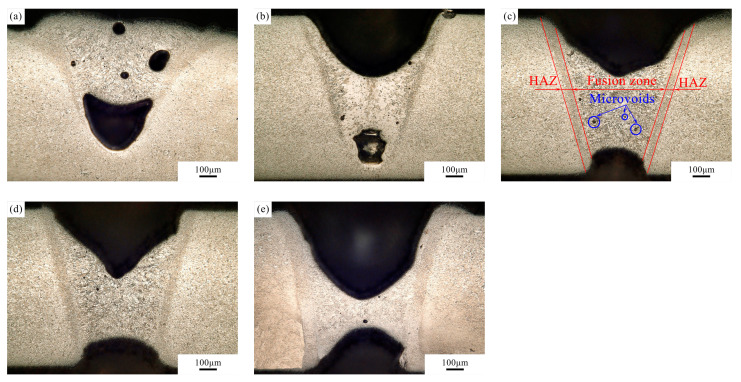
Optical micrographs of spots on the plate, with a power of 2500 W and a pulse duration of (**a**) 2 ms, (**b**) 2.5 ms, (**c**) 3 ms, (**d**) 3.5 ms and (**e**) 4 ms.

To study the effect of a pulsed laser beam on the microstructure of ODS Eurofer, a number of spots were created on a plate with varying parameters. A short melt pool lifetime would be beneficial for retaining the Y_2_O_3_ particles in the fusion zone. Therefore, a laser power of 2500 W and short pulse durations between 2 ms and 4 ms were applied. Optical micrographs of the cross-section of the spots can be seen in Figure 3. Material loss was observed in all conditions due to metal evaporation during the welding process. The width of the top of the “V”-shaped fusion zone is around 0.7 mm, which is very close to the beam size, indicating a concentrated heat input. The heat-affected zone (HAZ) is small in all cases, with a width of approximately 0.06 mm. It can be seen that partial penetration is obtained with a pulse duration of 2 ms and 2.5 ms. Large pores can be observed in the bottom of the fusion zone, probably because gas was trapped in the melt pool due to a short escape time. Full penetration is realised with pulse durations of more than 2.5 ms. As expected, more severe material loss was observed with longer laser beam pulses. Large pores managed to escape in these open keyhole conditions, while microvoids were found in the weld pool. 

### 3.2. Microstructure Characterisation

**Figure 4:**
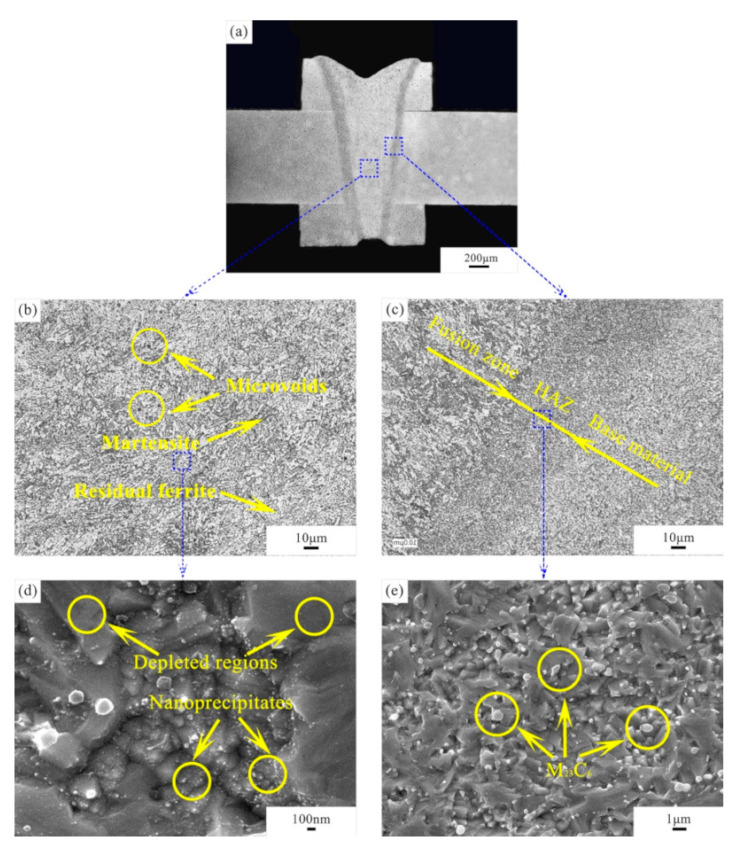
Morphologies of the weld seam with filler material: (**a**) SEM morphology of the weld seam, (**b**) fusion zone, (**c**) fusion line, (**d**) enlarged fusion zone and (**e**) enlarged HAZ.

In order to compensate for the material loss in the fusion zone, two ODS Eurofer square bars with dimensions of 30 × 1 × 0.5 mm^3^ were attached to the top and bottom surfaces of the work piece to act as a filler material. A duration of 5 ms was used to realise full penetration. The material was joined using the pattern indicated in Figure 2. An SEM image of the weld seam is shown in Figure 4a. It can be seen that the material loss in the specimen is fully compensated by the filler material. In Figure 4b, the microstructure has both martensite grains (dark regions) and ferrite grains (bright regions), with the presence of microvoids. Martensite is formed during the rapid cooling process (~10^4^–10^6^ K s^–1^ [15]), while δ-ferrite is formed during heating, as the peak temperature of laser welding is definitely higher than the austenite–δ-ferrite transformation temperature (around 1400 K). However, since the δ-ferrite–austenite transformation is a diffusion-controlled process, the rapid solidification does not offer sufficient time to complete the phase transformation [16]. Consequently, residual ferrite is observed in the microstructure.

**Figure 5:**
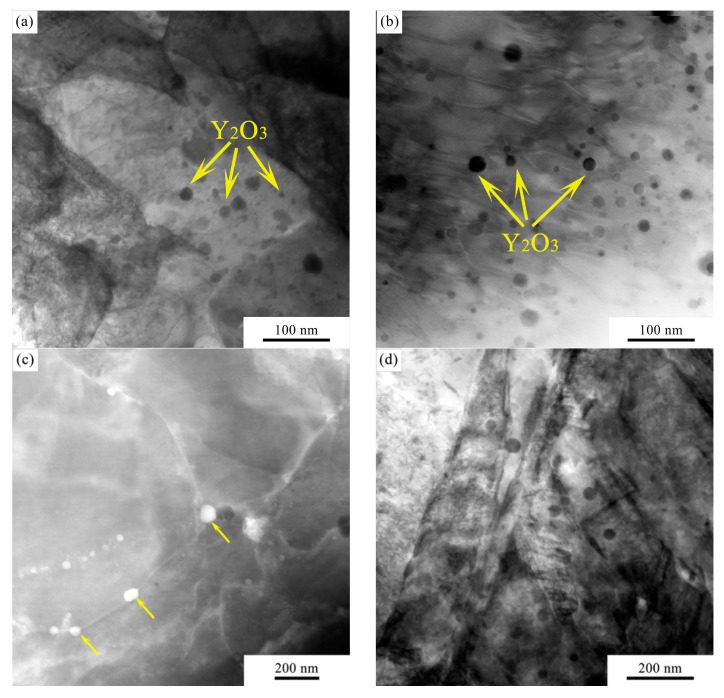
TEM images of the Y_2_O_3_ nanoparticles in the (**a**) base material, (**b**) fusion zone, (**c**) Y_2_O_3_ pinning the grain boundaries and (**d**) martensite lath.

Figure 4d shows an enlarged image of the microstructure in the fusion zone. A large number of nanoprecipitates can be observed in the steel matrix. As shown in the TEM images in Figure 5a,b, finely dispersed Y_2_O_3_ nanoparticles are observed in the microstructure. The particle sizes vary between 1 and 30 nm and do not show a significant difference in distribution between the fusion zone and base material. Figure 5c shows a dark field image of Y_2_O_3_ particles (indicated by the arrows) pinning the grain boundaries in the fusion zone, which is beneficial for enhancing the mechanical properties and extending the working temperature range. The martensite lath structure in the fusion zone is revealed in Figure 5d. From Figure 4d, it is also worth noting that the Y_2_O_3_ particles are not homogeneously distributed in the steel matrix. It seems that the smaller grains have a higher number of Y_2_O_3_ precipitates than the larger grains. This can be explained as follows: The distribution of Y_2_O_3_ is not perfectly homogenous even after a long period of mechanical alloying. Since Y_2_O_3_ nanoprecipitates have a strong effect on impeding grain growth through a Zener-type pinning [17], grains with a higher density of Y_2_O_3_ are presumably more resistant to recovery and growth.

The grain size of the HAZ is smaller compared to that of the base material and clearly smaller than that of the fusion zone (Figure 4c), probably due to martensite transformation. An enlarged image of the HAZ (Figure 4e) shows a large number of precipitates with a size ranging from 0.1 to 1 μm in the steel matrix. They are found to be more preferentially located at the grain boundaries, possibly due to a decrease in volume-free energy [17]. These precipitates are rich in Fe, Cr, W and C, which can be identified as M_23_C_6_ carbides (M = Fe, Cr and W) based on their size and chemical composition.

**Figure 6:**
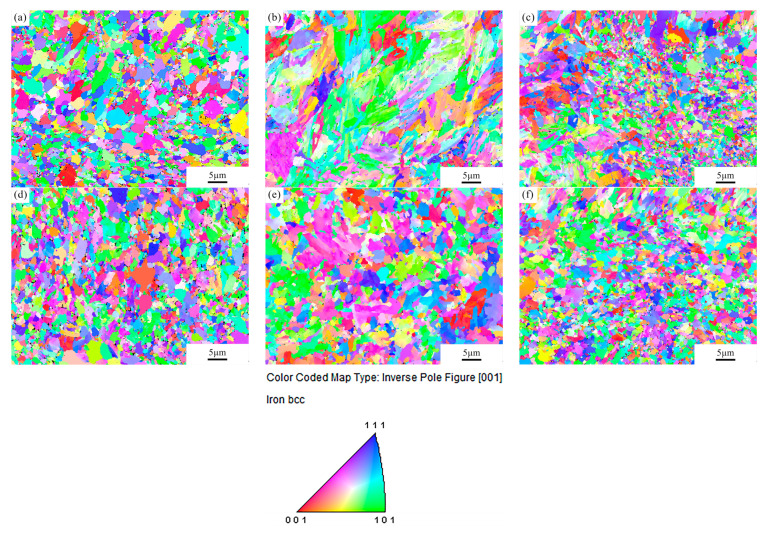
Inverse pole figure (IPF) maps of different regions: (**a**) base material, (**b**) fusion zone, (**c**) HAZ, (**d**) heat-treated base material, (**e**) heat-treated fusion zone and (**f**) heat-treated HAZ.

Figure 6 shows inverse pole figure (IPF) maps of the base material as well as the centre of the fusion zone and HAZ (P = 2500 W, t = 5 ms, with filler material) obtained by EBSD. Ultrafine grains smaller than 250 nm could not be indexed, causing the non-indexed (black) areas in the figures. It can be observed that the maps exhibit grain sizes ranging from the nanometre scale to a few micrometres. The microstructure of the fusion zone consists of elongated structures, while that of HAZ shows equiaxed grains. None of the regions show preferential grain orientation.

The average grain sizes were measured and are shown in Table 1. The grain size distribution was determined under the assumption that the minimum misorientation characterising grain boundaries is 15°. It can be seen that in the as-joined condition, the average grain size of different regions decreases from the fusion zone to the base material to the HAZ, which agrees with the observation from SEM. In the heat-treated condition, the average grain size of the fusion zone is still the largest, and that of the HAZ remains the smallest, but with smaller differences compared to the as-joined condition. Additionally, compared to the as-joined condition, there is a decrease in the grain size of the fusion zone, which could be ascribed to a refinement effect due to martensite transformation. Grain growth occurs in the HAZ, presumably resulting from the release of high stored energy due to phase transformation and distortion during laser welding. In general, the grain sizes of the joint overall do not grow significantly after the heat treatment, probably because of the strong pinning effect of Y_2_O_3_ on the motion of grain boundaries.

**Figure 7:**
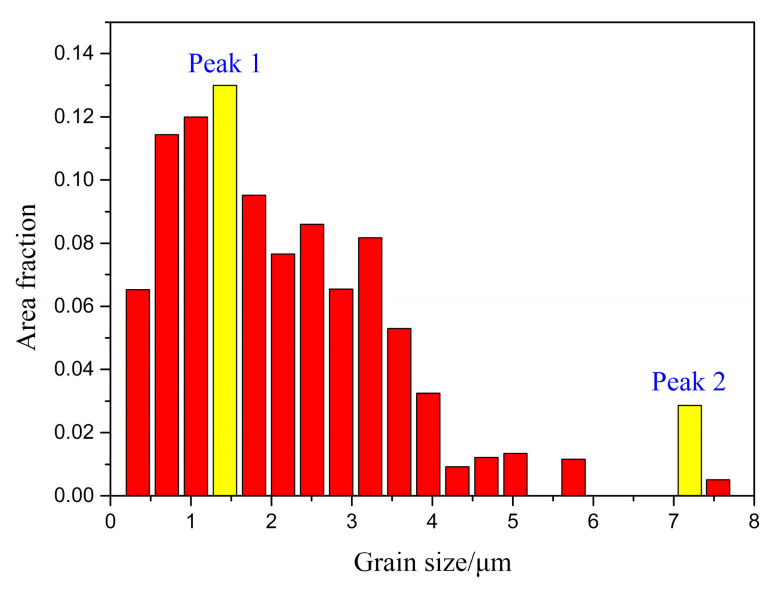
Grain size distribution of the fusion zone in the heat-treated joint (P = 2500 W, t = 5 ms).

**Figure 8:**
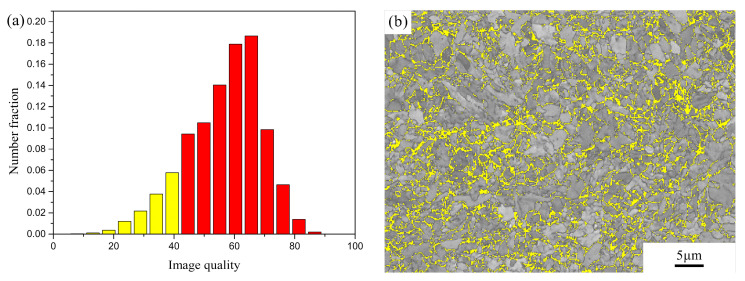
Fusion zone in the heat-treated joint (P = 2500 W, t = 5 ms): (**a**) Image quality in percentage showing highlighted low image quality and (**b**) Image quality map showing highlighted phases corresponding to martensite.

From Table 1, it can also be noted that the standard deviations of all conditions are high. This is due to a bimodal grain size distribution of the material. Taking the fusion zone in the heat-treated joint (Table 1) as an example, the grain size distribution has two peaks at 1.42 and 7.20 μm, respectively, as can be seen in Figure 7. Similar bimodal grain size distributions have been widely reported for powder metallurgy-prepared ODS steels [18,19,20]. In martensitic–ferritic steels, which generally contain 0.1–0.2 wt% C and 9–11 wt% Cr [21], this phenomenon could be due to the dual phase nature of the material. To further differentiate martensite from ferrite in the studied material, a chart of grain average image quality is shown in Figure 8a. Image quality (IQ) describes the quality of diffraction property of the analysed Kikuchi patterns. This factor can be used as an estimation of dislocation density or stored energy [22]. It can be seen that if a threshold value of approximately 40 is chosen, phases with image quality less than 40 are highlighted and shown in Figure 8b. In this way, martensite is differentiated from ferrite in the microstructure, since martensite exhibits lower image quality than ferrite due to its highly distorted lattice [23]. It can be noted that the smaller martensite grains are mainly located between the larger ferrite grains. This finding supports the dual phase microstructure of the material, and consequently, a high standard deviation of the grain size distribution.

**Figure 9:**
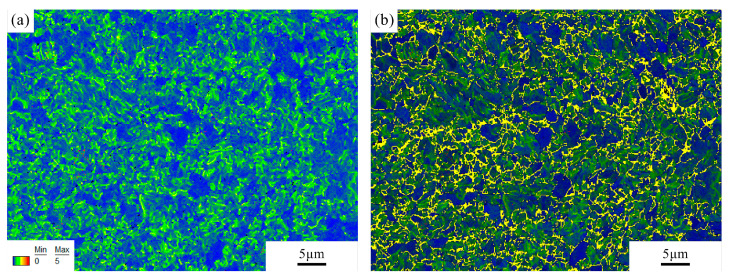
(**a**) Kernel average misorientation (KAM) map of the fusion zone in the heat-treated joint (P = 2500 W, t = 5 ms) and (**b**) Kernel average misorientation (KAM) map combined with image quality (IQ) map shown in Figure 8b.

**Figure 10:**
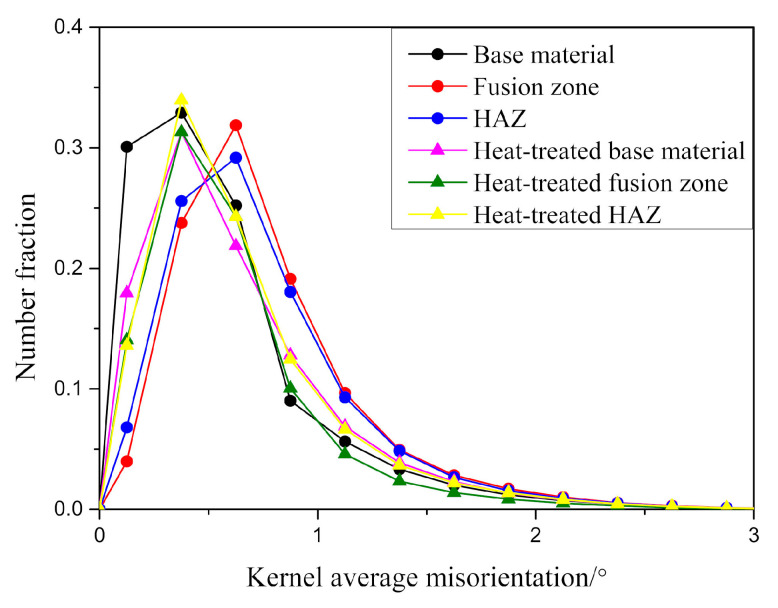
Distribution of kernel average misorientation (KAM) of different regions in the as-joined and heat-treated joints.

The kernel average misorientation (KAM), which represents the numerical misorientation average of a given point with its nearest neighbours, was used to characterise local misorientations. The maps were calculated using a maximum misorientation of 5°. Figure 9a shows the KAM map of the fusion zone in the heat-treated joint. It can be observed that the grains standing out from the rest due to a larger size show almost no misorientation, i.e., no local lattice distortion. In addition, misorientations between 0.5° and 2° are observed near the grain boundaries. These small grain regions with higher misorientations can be linked to the areas with lower image quality. As shown in Figure 9b, the KAM map combined with the IQ map reveals overlapping regions, where martensite grains (yellow) have a higher misorientation and ferrite grains (dark blue) generally have a lower misorientation. By comparing KAM of different regions in the joint, this indicates whether the microstructure is changed significantly after welding or the heat treatment. The fusion zone and HAZ have a higher KAM than the base material, indicating a more distorted microstructure and probably a higher martensite fraction due to an additional thermal cycle (Figure 10). Meanwhile, in the heat-treated condition, the KAM is smaller than that of the as-joined condition and shows no significant difference in different regions, demonstrating that the microstructure is substantially recovered and homogenous after the normalising and tempering treatment, which would be beneficial for the mechanical properties.

## 4. Discussion

With controlled welding conditions, including an optimised laser power and a short pulse duration, a fully penetrated and not overheated spot weld was obtained. The welding defects observed in the joint are mainly material loss and microvoids. The material loss is generally caused by metal evaporation and spattering. This could be improved by tuning the focal position and the divergence angle [24,25]. With a reduced power density around the keyhole aperture, the speed of the melt flow as well as the instability of the keyhole will be decreased; therefore, the evaporation and spattering phenomenon will be limited. As for the porosity defects, it has been found that porosity can be effectively inhibited at the optimum frequency and duty cycle of the employed laser pulse [26]. In addition, the keyhole stability and material evaporation also have a large effect on the generation of porosity [27]. The use of nitrogen instead of inert shielding gas was found to be effective to suppress the formation of porosity defects, due to the occurrence of the on-and-off cycle of nitrogen plasma prior to the initiation of keyhole instability [27]. All of these could be used to reduce the welding defects and thus, improve the mechanical performance of the weld in future works.

The melt pool lifetime during fusion welding is crucial for retaining the microstructure and mechanical properties of ODS steel joints. Upon welding, the oxide particles will quickly float to the top of the melt pool and, consequently, form a depleted area, leading to significantly reduced mechanical properties. With traditional “continuous” welding, the heat will accumulate in the weld. Even though techniques such as laser beam welding have a very high cooling rate, the melt pool lifetime is still too long for ODS steels. The temperature of the fusion zone usually stays above the melting point for a relatively long period of time. It is therefore difficult, if not impossible, to obtain an undisturbed microstructure, i.e., a microstructure with fine grains and dispersed nano-oxide particles. For instance, Lindau et al. [9] studied the joining of ODS Eurofer via electron beam welding, which also has a characteristic high power density. The results showed that the nano-dispersoids in the fusion zone unsurprisingly agglomerated to larger particles, causing a weak weld seam. Conversely, with the distributed pulsed spot welding procedure proposed in this study, the melt pool lifetime of each spot is reduced to the order of milliseconds, which is favourable for the retention of the microstructure and mechanical properties of the joint. It is known that the strengthening mechanisms of ODS steels are mainly based on grain boundary strengthening and dispersion strengthening [28], as neither the grain size nor the nanoparticle distribution is changed significantly after joining, and the strength of the joint will not be unduly reduced.

## 5. Conclusions

ODS Eurofer was welded successfully using a pulsed laser beam welding technique with a distributed pulse pattern. The welding parameters were optimised based on their effect on the microstructure. The microstructure of the joint in the as-joined and heat-treated conditions was investigated in detail. The main conclusions are as follows.

1. A full penetration is necessary to obtain an open keyhole condition without trapped gas. With a laser power of 2500 W, a pulse duration of at least 3 ms is needed for a 1 mm thick sample. A longer duration may lead to excess material loss, which can be compensated by the addition of filler materials.
2. In the as-joined condition, the fusion zone consists of elongated grains, while the HAZ has refined grains compared to the base material. M_23_C_6_ carbides are observed in the microstructure and found to be preferentially located at the grain boundaries. The nanoprecipitates are retained in the fusion zone, although they are not homogenously distributed in the steel matrix. Characterisations using small angle neutron scattering or small angle X-ray scattering could be carried out in the future to better understand the effect of laser beam welding on nanocluster behaviour.
3. EBSD results reveal that the joined material has a bimodal and dual phase microstructure. Martensite grains show a lower image quality and a higher misorientation compared to ferrite grains. The microstructure is generally recovered and homogenous after the heat treatment, which is beneficial for the mechanical properties. Our study shows that pulsed laser beam welding is an effective method for the joining of ODS Eurofer steel.

## Figures and Tables

**Table 1:** Average grain size of different regions obtained from EBSD data in Figure 6.

Conditions	Regions	Grain Size/μm
	Base material	2.08 ± 1.31
As-joined	Fusion zone	4.33 ± 2.89
	HAZ	1.64 ± 1.25
	Base material	1.92 ± 1.10
Heat treated	Fusion zone	2.20 ± 1.50
	HAZ	1.72 ± 1.07

## Data Availability

The raw/processed data required to reproduce these findings cannot be shared at this time as the data also form part of an ongoing study.
